# Catching a wave: On the suitability of traveling-wave solutions in epidemiological modeling

**DOI:** 10.1016/j.tpb.2024.12.004

**Published:** 2024-12-27

**Authors:** Anna M. Langmüller, Joachim Hermisson, Courtney C. Murdock, Philipp W. Messer

**Affiliations:** a Cornell University, Department of Computational Biology, 102 Tower Rd, Ithaca, 14850, NY, USA; b University of Vienna, Department of Mathematics, Oskar-Morgenstern-Platz 1, Vienna, 1090, Austria; c Aarhus University, Aarhus Institute of Advanced Studies, Høegh-Guldbergs Gade 6B, Aarhus C, 8000, Denmark; d Cornell University, Department of Entomology, 129 Garden Ave, Ithaca, 14850, NY, USA; e Max Perutz Labs, Vienna Biocenter Campus, Dr.-Bohr-Gasse 9, Vienna, 1030, Austria

**Keywords:** Ordinary differential equations, Diffusion theory, Spatial disease modeling, Individual-based simulations, SI/SIS/SIR model

## Abstract

Ordinary differential equation models such as the classical SIR model are widely used in epidemiology to study and predict infectious disease dynamics. However, these models typically assume that populations are homogeneously mixed, ignoring possible variations in disease prevalence due to spatial heterogeneity. To address this issue, reaction–diffusion models have been proposed as an alternative approach to modeling spatially continuous populations in which individuals move in a diffusive manner. In this study, we explore the conditions under which such spatial structure must be explicitly considered to accurately predict disease spread, and when the assumption of homogeneous mixing remains adequate. In particular, we derive a critical threshold for the diffusion coefficient below which disease transmission dynamics exhibit spatial heterogeneity. We validate our analytical results with individual-based simulations of disease transmission across a two-dimensional continuous landscape. Using this framework, we further explore how key epidemiological parameters such as the probability of disease establishment, its maximum incidence, and its final epidemic size are affected by incorporating spatial structure into SI, SIS, and SIR models. We discuss the implications of our findings for epidemiological modeling and identify design considerations and limitations for spatial simulation models of disease dynamics.

## 1. Introduction

A fundamental understanding of how infectious diseases spread through time and space is crucial for predicting the course of epidemics and guiding potential control measures. Compartmental models have been the workhorse for infectious disease modeling for almost a century (Kermack and McKendrick, 1927; Anderson and May, 1991; Murray, 2002). In these models, the population is divided into compartments based on their infection status, such as susceptible (S), infectious (I), and recovered (R) individuals. One of the simplest compartmental models is the so-called SI model, where individuals can only be susceptible or infected (Kermack and McKendrick, 1927). Once infected, an individual remains in that state forever. Two classical extensions of this model are the SIS and SIR models (Kermack and McKendrick, 1927): In the former, infected individuals can return to the susceptible class, while in the latter they can recover, often implying that they are immune to future infections.

Compartmental models are typically studied with ordinary differential equations (ODEs) that describe the expected changes in the proportions of the different population compartments over time (Kermack and McKendrick, 1927; Anderson and May, 1991; Hethcote, 1989, 2000; Murray, 2002). For example, consider a simple SI model where individuals come into contact with each other at a constant rate r. Let i(t) denote the fraction of infectious individuals in the population. Whenever a susceptible individual meets an infected one, it contracts the pathogen with probability α (for simplicity, we will use the terms ‘pathogen’ and ‘disease’ interchangeably throughout this paper, recognizing their conceptual distinction). Assuming homogeneous mixing among the individuals, the change in the proportion of infectious individuals over time is given by:

(1) $$\frac{\partial i}{\partial t}=r\alpha i(1-i)$$

For models with additional compartments, such as a recovered class in the SIR model, the dynamics are described by a set of coupled ODEs. Over the past century, such models have provided invaluable insights into the factors that shape disease transmission by providing conceptual results and threshold quantities (e.g., herd immunity, basic reproduction number, and replacement number) to predict disease spread and the impact of countermeasures, thereby guiding decision-making and public health policy during disease outbreaks (Hethcote, 1989; Anderson and May, 1991; Murray, 2002; Hethcote, 2000; López-Flores et al., 2021).

The limitations of ODE models often lie in the underlying assumptions they make about the transmission process. A common assumption of ODE models is homogeneous mixing among individuals (Grassly and Fraser, 2008). This is rarely the case for real-world populations, which can be structured in many ways (Lewis and Kareiva, 1993; Mollison, 1991; Grenfell and Dobson, 1995; Riley, 2007; Brockmann and Helbing, 2013). In particular, most populations are geographically structured, so that individuals living close together are more likely to meet than individuals living far apart. Besides spatial structure, kinship and social structure can also have a significant impact on contact patterns (Rozins et al., 2018; Buckee et al., 2021). As a result, local disease prevalence can vary substantially between different parts of the population, leading to notable differences in the expected epidemiological dynamics (Capasso, 1993; Riley, 2007; Riley et al., 2015). While classical ODE models have clear advantages for modeling disease transmission, such as their well-understood mathematical properties, computational efficiency, and ability to provide analytical solutions (Riley, 2007), ignoring the influence of population structure in these models can lead to inaccurate conclusions.

In this study, we focus on the potential effects of spatial population structure, which can be incorporated into disease transmission models in several ways. One approach is to use a meta-population model that divides the population into subpopulations or “patches”, each representing a homogeneous population such as a city. The dynamics within the patches, and the coupling between them, can then be described by a system of ODEs (Brauer et al., 2019; Lajmanovich and Yorke, 1976; Belik et al., 2011). Such coupling could be based on geographic distance, empirical observations such as mobile phone data (Wesolowski et al., 2015), or geolocated social media posts (Blanford et al., 2015). Alternatively, individual- or agent-based approaches can be used to explicitly model all individuals in the population and their spatial location (Perkins et al., 2019; Riley et al., 2015; Reiner et al., 2014). The interactions between individuals (e.g., the rate and strength of contacts) can be regulated by distance-based kernels (Hethcote, 2000; Ferguson et al., 2001), which can result in highly variable infection probabilities across space (Riley, 2007). Network models represent another class of approaches that allow for fine-scale modeling of population structure (Keeling and Eames, 2005). In these models, each node typically represents an individual, with edges between nodes representing contacts between individuals. A susceptible individual can only contract the disease if an edge connects it to an infectious individual (Riley et al., 2015; Rushmore et al., 2013). However, network models are complex, and their analysis may require a large amount of empirical data (Riley et al., 2015).

For populations inhabiting a continuous landscape, where individuals move primarily within short distances relative to the total geographic range, reaction–diffusion models offer an alternative approach to describe spatial disease dynamics (Shigesada and Kawasaki, 1997; Hotelling, 1921). These models gained popularity in the 1930s, when both Fisher and Kolmogorov used them to explain the spread of a beneficial allele in a one-dimensional habitat (Fisher, 1937; Kolmogorov et al., 1937). In such models, the beneficial allele can propagate through the population as a “traveling wave”, with a constant and well-defined velocity. Since then, diffusion models have been widely used in mathematical biology to study not only the spread of adaptive alleles but also invasive organisms and infectious diseases (Hotelling, 1921; Wang, 2014; Noble, 1974; Shigesada and Kawasaki, 1997; Mundt et al., 2009; Smith et al., 2002; Novembre et al., 2005; Hastings et al., 2005; Chen, 2014; Murray, 2002; Steiner and Novembre, 2022). In the context of epidemiology, reaction–diffusion models describe the proportion of infected individuals as a function of space and time. The diffusion term models the random dispersal of infectious individuals or contacts (Mollison, 1972), while the reaction term describes the local rates of disease transmission, similar to a compartmental ODE model (Shigesada and Kawasaki, 1997).

In this paper, we use diffusion theory to study under which scenarios a simple compartmental ODE model is sufficient to capture the disease dynamics and when spatial structure needs to be considered. We derive a simple critical threshold $D_{c}$ for the diffusion coefficient, below which the limited dispersal of individuals significantly affects the disease dynamics. To validate our findings, we perform individual-based simulations using SLiM (Haller and Messer, 2023), which allow us to smoothly transition from spatially unstructured to structured populations. We examine the effect of spatial structure on disease dynamics in three classical compartmental transmission models: the SI, SIS, and SIR model (Kermack and McKendrick, 1927; Hethcote, 1989). Furthermore, we highlight potential caveats of simulating continuous disease transmission models in a discrete, individual-based, and biologically realistic manner.

## 2. Results

### 2.1. ODE model

Consider a simple Susceptible-Infected (SI) model in a closed population of constant size N without birth or death events (Kermack and McKendrick, 1927; Hethcote, 1989). There are only susceptible and infectious individuals in the population, and infectious individuals do not recover (i.e., they never leave the infectious compartment). Let I(t) and S(t)=N-I(t) denote the total number of infectious and susceptible individuals in the population at time t, and s(t)=S(t)/N and i(t)=I(t)/N their respective population frequencies with i(t)+s(t)=1. If we assume a homogeneously mixing population in which individuals come into contact with each other at a constant contact rate r per time unit, and a disease that establishes in a susceptible individual after contact with an infectious individual with probability α, then the change in the proportion of infectious individuals is given by Eq. (1). The solution to this ODE model is a logistic growth function (Tsoularis and Wallace, 2002):

(2) $$i(t)=\frac{1}{1+[N/I(0)-1]e^{-r\alpha t}}$$

**Fig. 1.**
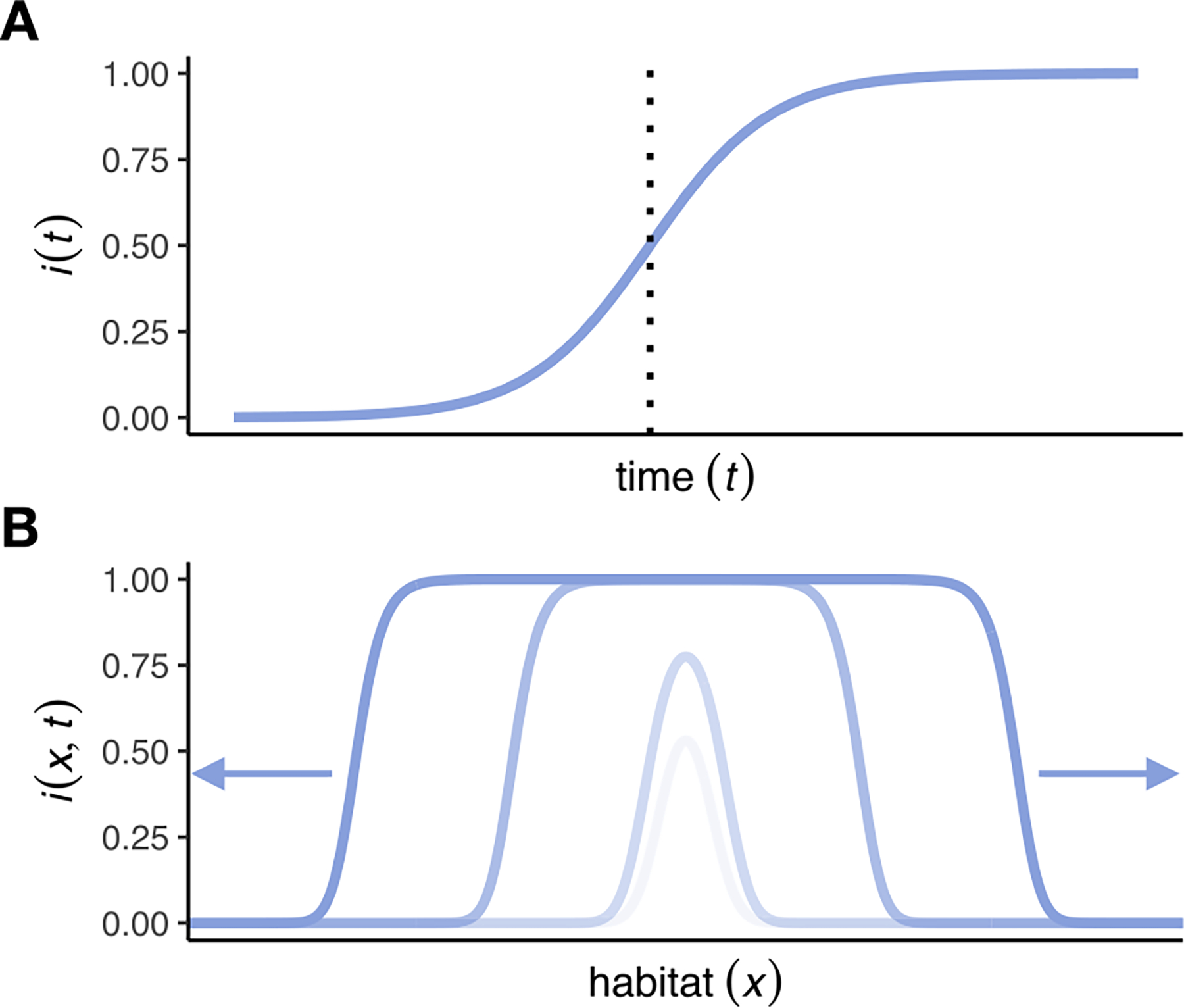
Schematic plots of expected disease spread under the ODE model and a traveling wave solution of the reaction–diffusion model. (A) In the ODE model, the frequency of infected individuals, i(t), increases logistically according to Eq. (2). The vertical dashed lines represent $t_{1/2}$ as defined in Eq. (3). (B) In the diffusion model for a one-dimensional habitat, the disease spreads from an initial release at the center of the habitat via two traveling waves with minimum velocity $c_{0}=2\sqrt{Dr\alpha }$ in either direction.

Whenever I(0)>0, we have ${\text{lim}}_{t\to \infty }i(t)=1$, meaning that the disease will eventually spread throughout the entire population. Fig. 1A provides an illustration of this logistic spread in the ODE model. Note that the parameters r and α always appear together, with the composite parameter $(r\alpha {)}^{-1}$ setting the timescale of the dynamics. However, since r and α regulate different processes in the context of our individual-based simulation framework described in Section 2.4, we do not combine them into one composite variable.

Assuming a single infected individual at time t=0(I(0)=1) and a sufficiently large population (N≫1), the expected time for the disease to reach 50% frequency in the population is:

(3) $$t_{1/2}=\frac{\text{ln}[N-1]}{r\alpha }\approx \frac{\text{ln}\;N}{r\alpha }$$

Since i(t) is S-shaped with the largest growth rate at the inflection point $i\left(t_{1/2}\right)=1/2$, the expected time for the disease to spread through the whole population, under the assumptions N≫1 and I(0)=1, is therefore:

(4) $$t_{\text{ODE}}\approx 2t_{1/2}\approx \frac{2\;\text{ln}\;N}{r\alpha }$$

We call $t_{\text{ODE}}$ the “fixation time” of the ODE model.

### 2.2. Reaction–diffusion model

In contrast to the classical ODE-based compartmental models described above, reaction–diffusion models provide an alternative that explicitly incorporates spatial heterogeneity in disease prevalence (Murray, 2002; Scherm, 1996; Hastings, 1996; Fisher, 1937; Kolmogorov et al., 1937; Hotelling, 1921). In a diffusion model, the frequency of infected individuals becomes a function not only of time t but also of spatial position x.

Consider an SI model in which individuals move randomly according to a dispersal kernel that decays with distance at least as quickly as an exponential function (Steiner and Novembre, 2022). In such a case, we can model dispersal by a diffusion term (Steiner and Novembre, 2022; Fisher, 1937; Shigesada et al., 1995; Hotelling, 1921), while disease transmission can be modeled by a reaction term based on the local compartment frequencies. Together, this yields a second-order partial differential equation for the SI diffusion model of the form:

(5) $$\frac{\partial }{\partial t}i(x,t)=D\frac{\partial^{2}}{\partial x^{2}}i(x,t)+r\alpha i(x,t)[1-i(x,t)]$$

The diffusion coefficient D accounts for the random dispersal of individuals. In a one-dimensional habitat, $2D=\sigma^{2}$, where $\sigma^{2}$ is the variance in the expected spatial displacement of individuals per time unit due to their random movement (Fisher, 1937; Tkachenko et al., 2017). The reaction term is analogous to the ODE model from Eq. (1), except that the frequency of infected individuals now depends on the spatial position x as well. Eq. (5) is also known as the Fisher–Kolmogorov or Fisher–KPP equation (Fisher, 1937; Kolmogorov et al., 1937; Murray, 2002).

We can express Eq. (5) in dimensionless form by rescaling time, $\tilde{t}=(r\alpha )t$, and space, $\tilde{x}=x/(\sqrt{D/(r\alpha )})$ where $(r\alpha {)}^{-1}$ is the characteristic timescale of the system:

(6) $$\frac{\partial }{\partial \tilde{t}}i(\tilde{x},\tilde{t})=\frac{\partial^{2}}{\partial {\tilde{x}}^{2}}i(\tilde{x},\tilde{t})+i(\tilde{x},\tilde{t})[1-i(\tilde{x},\tilde{t})]$$

However, in the context of our individual-based simulation model (Section 2.4), it is more convenient to maintain the habitat size L and the simulation step-size (i.e., “tick”) Δt as the units of space and time. Thus, we retain the parameterization of the Fisher–KPP equation in (5) to facilitate the mapping between the continuous diffusion model and the discrete simulation framework.

Under the assumption that the sum of susceptible and infectious individuals is conserved at a constant carrying capacity, and that there is no change in dispersal due to infection, one solution to Eq. (5) is a so-called “traveling wave” (Fisher, 1937; Fife, 1979; Shigesada and Kawasaki, 1997; Murray, 2002; Steiner and Novembre, 2022; Mundt et al., 2009; Källén, 1984; Wang and Wang, 2016) in which the disease spreads outward from an initial introduction point. The minimum velocity $c_{0}$ of this wave is:

(7) $$c_{0}=2\sqrt{Dr\alpha }$$

Fig. 1B provides an illustration of these dynamics in a one-dimensional habitat, where the disease is introduced at the center and then spreads outward in the form of two symmetric traveling waves to the left and right. The “width” of each wave, i.e., the length of the region between its front (where the frequency of infected individuals is still close to zero) and top (where almost everyone is already infected), is approximately $w\approx 2\sqrt{D/(r\alpha )}$ (Fisher, 1937).

It is straightforward to extend this diffusion model to two dimensions. Specifically, if dispersal is isotropic, the displacement along the x and y axes can be treated independently, with each following the one-dimensional model. The diffusion coefficient then equals half the variance of the displacement in each of the x and y coordinates: $2D=\sigma_{x}^{2}=\sigma_{y}^{2}$. Note that the total displacement in a two-dimensional space, $\Delta =\sqrt{\Delta_{x}^{2}+\Delta_{y}^{2}}$, will typically be larger than the displacement in each individual dimension.

In this two-dimensional diffusion model with isotropic dispersal, the disease spreads from an initial introduction point in the form of a growing circle. As the curvature of the wavefront decreases, the velocity at which the radius grows asymptotically approaches the same minimum wave speed $c_{0}$ as the traveling wave in the one-dimensional model (Lewis and Kareiva, 1993; Zhuang and Wang, 2021). If the disease is introduced in a small number of individuals in the center of a square habitat patch with a side length L that is much larger than the wave width (which requires $L\gg 2\sqrt{D/(r\alpha )}$ (Kot, 2001)), the wave reaches the corners of the square patch when the circle grows to a radius of $L/\sqrt{2}$.

After an initial phase to establish a wavefront, the expected fixation time of the SI diffusion model is thus governed by the travel time of the wave to reach the corners of the patch, which is approximately:

(8) $$t_{\text{DIFF}}\approx \frac{L}{\sqrt{8Dr\alpha }}$$

This expression only accounts for the spatial progression and ignores the time it takes for the wavefront to build up and the time for full fixation after the front reaches the habitat boundaries. These are local growth processes. As a simple heuristic, we approximate the additional time needed for these phases by the time for logistic growth of the corresponding ODE model and thus set $t_{\text{fix}}=t_{\text{DIFF}}+t_{\text{ODE}}$ for the total fixation time. More generally, we approximate the time for the disease to reach a given frequency i(t) in the total population by the sum of the expected time to reach that frequency under a traveling wave Eq. (7) and the time required under logistic growth Eq. (2) alone. We test this heuristic in the simulation below.

### 2.3. Critical threshold

We can now ask under what conditions the spatial component of the disease dynamics cannot be ignored, so that an approximation by the simple ODE model fails. Our exposition of the expected disease fixation time suggests a simple criterion: The spatial component of the dynamics dominates if the travel time of the wave in the diffusion model, $t_{\text{DIFF}}$, (8), exceeds the time required for homogeneous logistic growth, $t_{\text{ODE}}$, (3). Setting both equal, we obtain a critical value for the diffusion coefficient:

(9) $$t_{\text{ODE}}=t_{\text{DIFF}}⟹D_{c}=\frac{r\alpha L^{2}}{32(\text{ln}\;N{)}^{2}}$$

When $D<D_{c}$ the slow dispersal of individuals through the habitat becomes the limiting factor for the spread of the disease. In this low-dispersal regime, we expect the diffusion model to describe the epidemiological dynamics more accurately than the ODE model, which overestimates the rate of spread.

As D becomes of the order of $D_{c}$, the width of the traveling wave approaches the habitat size, and the disease no longer spreads as a circle with a “sharp” edge (Kot, 2001). Spatial progression ceases to limit the disease spread, and the fixation time should gradually approach the prediction of the ODE model. In the high-dispersal regime where $D\gg D_{c}$, diffusion is strong enough that the population is effectively well mixed over the timescale relevant for disease spread, and the ODE model should describe the epidemiological dynamics accurately.

### 2.4. Individual-based simulation framework

To test the theoretical predictions derived above, we implemented an individual-based distributed-infectives simulation model for disease transmission in SLiM (version 4.0) (Haller and Messer, 2023). By varying the dispersal rates of individuals, our simulation model can smoothly transition from spatially unstructured to highly structured populations, allowing us to investigate disease dynamics across the high and low-dispersal regimes, and to study the predicted transition around the critical diffusion coefficient $D_{c}$. We focused on an abstract, directly transmitted disease in a closed population (i.e., no births or deaths) of N=100,000 individuals. These individuals move in a continuous two-dimensional spatial domain, modeled as a square habitat patch with a side length of 1 (i.e., L=1) and periodic boundaries to avoid edge effects (Mazzucco et al., 2018; Capasso, 1993).

The ODE and diffusion models are both continuous time models. Our individual-based simulation model in SLiM, on the other hand, measures time in discrete units of “ticks” (i.e., Δt=1). Each such “tick” is associated with the opportunity for new infections and recovery of individuals. To minimize differences between the discrete-time simulations and the continuous-time mathematical models, we must ensure that Δt is sufficiently small compared to the characteristic timescale $(r\alpha {)}^{-1}$. However, this requirement does not limit the generality of our models. We can freely choose the real-world time interval that a tick in our simulations corresponds to, be it a year or a millisecond. By choosing a sufficiently small period, we can always ensure that $\Delta t\ll (r\alpha {)}^{-1}$.

The disease is transmitted via local contacts between infectious and susceptible individuals. In each tick, we assume that an individual has contact with all individuals that are currently within its interaction distance δ, which we chose so that each individual has on average 15 contacts per tick (i.e., r=15). Note that the average number of contacts per tick is independent of the infection status (i.e., infectious individuals have, on average, as many contacts as susceptible or recovered individuals). For a square habitat with edge length normalized to L=1 and toroidal boundary conditions, and with 100,000 individuals, this corresponds to an interaction radius of δ≈0.00691. Any contact between a susceptible individual and an infectious individual will result in disease transmission with a probability of α=0.001, giving $(r\alpha {)}^{-1}\approx 66.67$ ticks ≫1. Once a susceptible individual has contracted the disease, it becomes infectious in the next tick. At the end of each tick, we simulate isotropic dispersal by sampling the x and y displacements for each individual independently from a normal distribution with mean μ=0 and variance $\sigma^{2}=2D$. The infection status of an individual has no effect on D.

We implemented three compartmental disease transmission models in this simulation framework: (i) the SI model, (ii) the SIS model, and (iii) the SIR model (Hethcote, 2000). In the SI model, once an individual is infected, it remains infectious until the end of the simulation. In the SIS model, infected individuals can recover and return to the susceptible class in each tick with probability γ. In the SIR model, they recover and gain permanent immunity with probability γ in each tick.

Each simulation run is initialized by uniformly distributing 100,000 susceptible individuals throughout the habitat. At tick 25, the disease is introduced into the population by infecting the individual closest to the center of the square habitat (i.e., I(0)=1). For each simulation, we recorded the number of individuals in each compartment at the beginning of each tick until either the disease spread through the whole population, no infectious individuals were left, or the simulation ran for 50,000 ticks. This upper threshold of 50,000 ticks results in a very mild constraint that is not expected to interfere with the simulated dynamics of SI disease transmission. For our simulation parameters, the disease is expected to fix in the SI model between approximately 1500–14,000 ticks Eqs. (4) and (10).

#### 2.4.1. Low-diffusion limit

A key abstraction of the diffusion approach is that it models a continuous density of individuals throughout the habitat. The relative frequencies of infectious and susceptible individuals are specified at any given location in the habitat, and disease transmission can occur exactly at that point. In our individual-based simulation model, however, a finite number of individuals are distributed across a continuous habitat. It is unlikely that any two of them will ever be at exactly the same point. Thus, one has to decide how close two individuals must be for one to be able to infect the other, which we have implemented by defining an interaction radius δ.

Ideally, δ should be very small (i.e., much smaller than the average dispersal distance of individuals) so that the movement of individuals remains the primary driver of disease spread. Yet, there is a lower limit on δ for any given contact rate in our individual-based simulation model. For example, if we want to ensure that each individual has, say, two contacts on average, this requires some minimum interaction radius so that each individual encounters enough individuals within its interaction radius. In our simulated continuous two-dimensional habitat, which is represented by a square with a side length of L and containing N uniformly distributed individuals, the interaction radius δ is defined as $\delta =L\sqrt{r/(N\pi )}$.

One important consequence is that, when D becomes sufficiently small, the spread of the disease is no longer driven primarily by the dispersal of individuals, but instead by “hopping” between neighboring individuals with overlapping interaction circles. These “hopping” dynamics can cause the disease to fix even if individuals do not move at all, thus setting an upper bound for the fixation time in the limit D→0.

We can still describe disease spread under these hopping dynamics by the diffusion model, but we need to reinterpret the diffusion term. In particular, we interpret each transmission event as a “dispersal” step of the disease from the location of the infecting individual to the location of the newly infected individual. To derive a rough estimate of the diffusion coefficient ($D_{0}$) of the transmission, we consider the limit where individuals no longer move at all. Starting from the first infected individual at the center of the habitat, the disease spreads outward in a circular pattern, with the traveling wave now driven entirely by transmission events rather than by individual dispersal. The minimum speed of this traveling wave for the SI model is given by $c_{0}=2\sqrt{D_{0}r\alpha }$ (Källén, 1984; Wang and Wang, 2016). One complication is that, in the hopping model, $D_{0}$ varies in space because it depends on the occurrence of new transmission events. Thus, $D_{0}$ approaches zero in areas where either everyone is already infected or everyone is still susceptible. It is maximal right at the wavefront, where it determines the speed of the wave.

Let us, therefore, consider an infected individual located exactly at the wavefront. If its neighbors are uniformly distributed across its interaction circle, about half of these individuals are (on average) already infected, while the others are still susceptible. The overall rate at which transmission events from the focal infected individual occur then is Pr(transmission)=rα/2. When a transmission event occurs, the variance of the displacement along the x axis is $\sigma_{x}^{2}=\delta^{2}/4$. The effective diffusion coefficient of the hopping process thus results as $2D_{0}\approx \text{Pr}(\text{transmission})\sigma_{x}^{2}=r\alpha \delta^{2}/8$. According to Eq. (8), this yields a fixation time of:

(10) $$t_{0}\approx \frac{\sqrt{2}L}{r\alpha \delta }$$

This provides an approximate upper bound for the fixation time in the limit D→0, where the fixation time from dispersal alone, given by Eq. (8), diverges to infinity. By comparing the fixation time estimates $t_{0}$ and $t_{\text{DIFF}}$, we determine $D_{l}$, a threshold for individual dispersal below which disease spread in our individual-based simulation model is primarily driven by transmission events:

(11) $$t_{0}=t_{\text{DIFF}}⟹D_{l}=\frac{r\alpha \delta^{2}}{16}=\frac{(rL{)}^{2}\alpha }{16N\;\pi }.$$

### 2.5. SI model

**Fig. 2.**
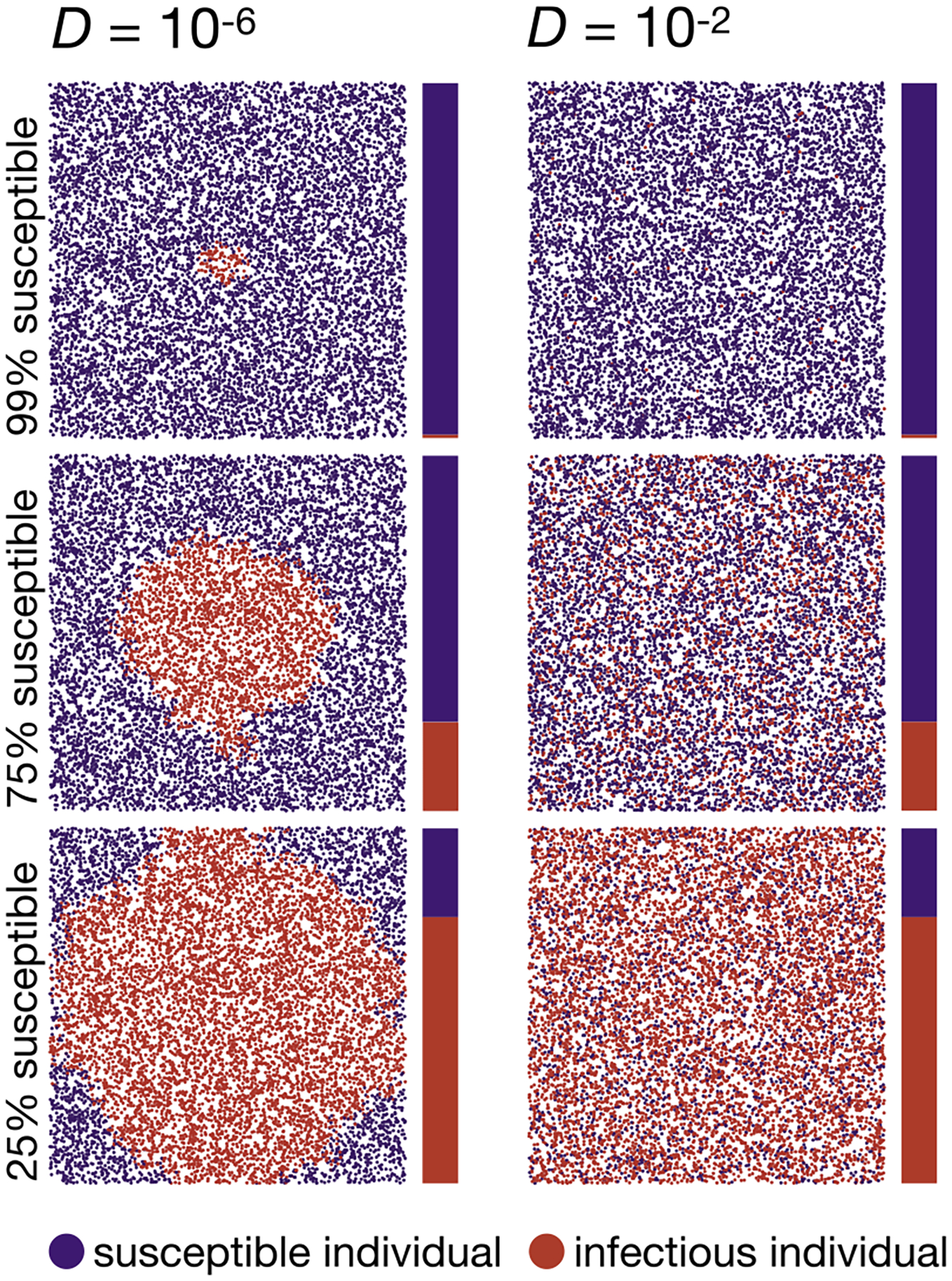
Two example runs of the SI simulation model. The left column shows a run in the low-dispersal regime ($D=10^{-6}$), while the right column shows a run in the high-dispersal regime ($D=10^{-2}$). The top, middle, and bottom plots show population snapshots taken when 99%, 75%, and 25% of the population were still susceptible. As predicted, the disease progresses in a circular pattern in the low-dispersal regime. In contrast, with high dispersal, infectious individuals are homogeneously distributed in space. Data were simulated using N=10,000 (ten times smaller than our standard model for better visualization), α=0.01,r=15,L=1. Videos of simulation runs in the SI model can be found under https://tinyurl.com/bdddam58.

To test how well our mathematical predictions describe disease spread in the individual-based simulations, we start with the SI model. Qualitatively, we expect that in the low-dispersal regime ($D<D_{c}=3.536\times 10^{-6}$), the disease spreads from its introduction at the center of the habitat as a circle with a steadily growing radius. By contrast, in the high-dispersal regime ($D\gg D_{c}$), the population should be sufficiently mixed so that the frequency of infected individuals increases in all areas of the habitat at a similar rate. Fig. 2 confirms these qualitative predictions when comparing two simulation runs with $D=10^{-6}$ (left column) and $D=10^{-2}$ (right column).

**Fig. 3.**
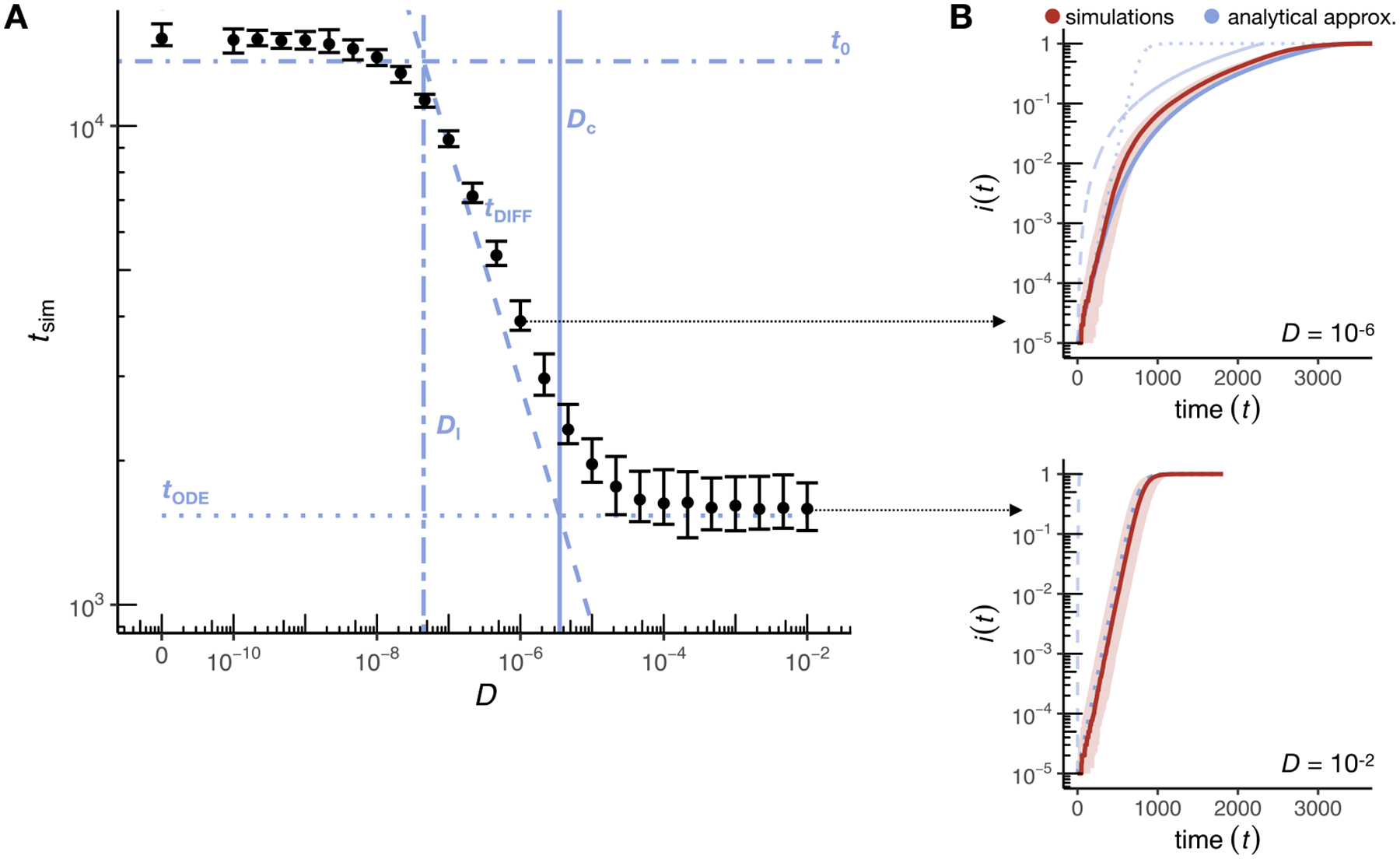
Disease dynamics in the SI model. (A) Time until disease fixation in the simulations, $t_{\text{sim}}$, for varying diffusion coefficients D. Black dots represent median values and error bars the 2.5 and 97.5 percentiles estimated from 50 simulation runs for each D (for D=0, the spread stagnated in 3 of the 50 runs due to small clusters of susceptible individuals with interaction areas without infectious individuals, which we excluded from the analysis). The vertical solid line indicates the critical threshold $D_{c}$ according to Eq. (9). In the high-dispersal regime ($D\gg D_{c}$), fixation times converge to the prediction of the ODE model ($t_{\text{ODE}}$, dotted line), while in the limit of no dispersal (D→0) they are bounded by the prediction of the hopping model ($t_{0}$, dot-dashed line; $D_{l}$ is indicated as a vertical double-dashed line). In the low-dispersal regime where $D<D_{c}$, the disease advances as a traveling wave, and $t_{\text{sim}}$ is well approximated by $t_{\text{DIFF}}$ (dashed line). (B) The upper panel shows the time-resolved disease dynamics for the low-dispersal regime ($D=10^{-6}$). Here, the proportion of infectious individuals, i(t), increases after an initial lag phase approximately as the area of a circle whose radius expands at the predicted minimum wave speed of the diffusion model (blue dashed line). The initial lag between the expected and the observed proportion of infectious individuals is likely caused by the local establishment of the disease and an initial reduction in wave speed due to the curvature of the wavefront (Lewis and Kareiva, 1993; Shigesada et al., 1995). The i(t) of the simulations is well approximated by the heuristic approximation $t_{\text{fix}}$ (solid blue line) that accounts for both, the disease propagation via a constant traveling wave, and the local rise in i(t) described by the ODE model (dotted line). The bottom panel shows an example from the high-dispersal regime ($D=10^{-2}$). Here, the fraction of infectious individuals is well approximated by the logistic growth function of the ODE model (dotted blue line). Solid red lines represent the median across 50 simulation runs and the shaded areas represent the range between the 2.5 and 97.5 percentiles. All data were simulated using N=100,000,α=0.001,r=15,L=1.

We next tested our predictions for the disease fixation time under varying dispersal rates (Fig. 3A). In the high-dispersal regime, the observed fixation time $t_{\text{sim}}$ is independent of D and well approximated by the predictions from the ODE model given in Eq. (4). The time-resolved proportion of infectious individuals follows the logistic growth curve (Fig. 3B, bottom panel). When D becomes smaller than $D_{c}$, the fixation time starts to increase and becomes inversely proportional to D, as predicted by Eq. (8). Once D becomes smaller than $D_{l}$, the dispersal threshold below which disease spread in our individual-based simulation model is primarily driven by transmission events ($D_{l}=4.476\times 10^{-8}$), the fixation time approaches the prediction under the hopping dynamics derived in Eq. (10), $t_{\text{sim}}$ levels off, and again becomes independent of D.

Overall, we observe that fixation times can differ up to one order of magnitude in our simulation model depending on the dispersal rate. This highlights the risk of underestimating the expected epidemic duration if predictions are based solely on the ODE model without accounting for population structure.

### 2.6. SIS model

In the SI model, infected individuals do not recover from the infection, leading to an inevitable spread and eventual fixation of the disease as long as rα>0. The SIS model allows infected individuals to transition from infection back to the susceptible state at rate γ. In a deterministic ODE model of an unstructured population, such a disease is expected to approach a constant frequency of $1-1/R_{0}$ for $R_{0}>1$, where $R_{0}=r\alpha /\gamma$ represents the basic reproduction number of the disease (i.e., the average number of secondary infections caused by a single infectious individual when introduced into a completely susceptible population) (Dietz, 1993; Hethcote, 1989). If $R_{0}<1$, the disease is unable to become established in the population and will typically die out quickly. Much work has been done to define $R_{0}$ in reaction–diffusion models, which are inherently more complex than their ODE counterparts (Yang and Wang, 2023). Although the $R_{0}$ definitions in reaction–diffusion models differ from the classical ODE-based definition, they can converge under certain conditions, such as when diffusion rates approach 0 (Chen and Shi, 2020; Yang and Wang, 2023). In this study, we use the classical ODE-based $R_{0}$ as a reference point for easier comparison across different dispersal regimes.

Analogous to the SI model, one can again determine a critical threshold $D_{c}$, below which individual dispersal is expected to affect the disease dynamics. For the SIS model, the frequency of infected individuals i(t) follows a logistic growth function with a carrying capacity of $1-1/R_{0}=1-\gamma /(r\alpha )$ in a homogeneously mixing population. With I(0)=1,

(12) $$i(t)=\frac{1-\gamma /(r\alpha )}{1+(N(1-\gamma /(r\alpha ))-1)e^{-(r\alpha -\gamma )t}}$$

Eq. (12) allows the derivation of an expected “time until constant endemic frequency” estimate for the ODE model, similar to the derivation of the “fixation time” estimate for the SI model Eq. (4). Assuming N≫1, this “time until constant endemic frequency” can be estimated for the SIS model in a homogeneously mixing population as:

(13) $$t_{\text{ODE}}\approx 2t_{1/2}\approx \frac{2\;\text{ln}[N(1-\gamma /(r\alpha ))-1]}{r\alpha -\gamma }\approx \frac{2\;\text{ln}[N(1-\gamma /(r\alpha ))]}{r\alpha -\gamma }$$

For a spatial SIS model, the recovery of infectious individuals back to the susceptible state leads to a slowing of wave propagation, with the recovery rate γ entering the minimum wave speed approximation as $c_{0}=2\sqrt{D(r\alpha -\gamma )}$ (Fisher, 1937; Källén, 1984; Wang and Wang, 2016). We thus obtain the time required for the spatial spread of the disease in the SIS model as:

(14) $$t_{\text{DIFF}}\approx \frac{L}{\sqrt{8D(r\alpha -\gamma )}}$$

Following the lines discussed above for the SI model, we can compare the expected time to constant endemic frequency, $t_{\text{ODE}}$, in the ODE model with the spread time, $t_{\text{DIFF}}$, to obtain a critical value for the diffusion coefficient above which we expect a negligible effect of spatial progression on disease dynamics:

(15) $$t_{\text{ODE}}=t_{\text{DIFF}}⟹D_{c}=\frac{(r\alpha -\gamma )L^{2}}{32(\text{ln}[N(1-\gamma /(r\alpha ))]{)}^{2}}$$

**Fig. 4.**
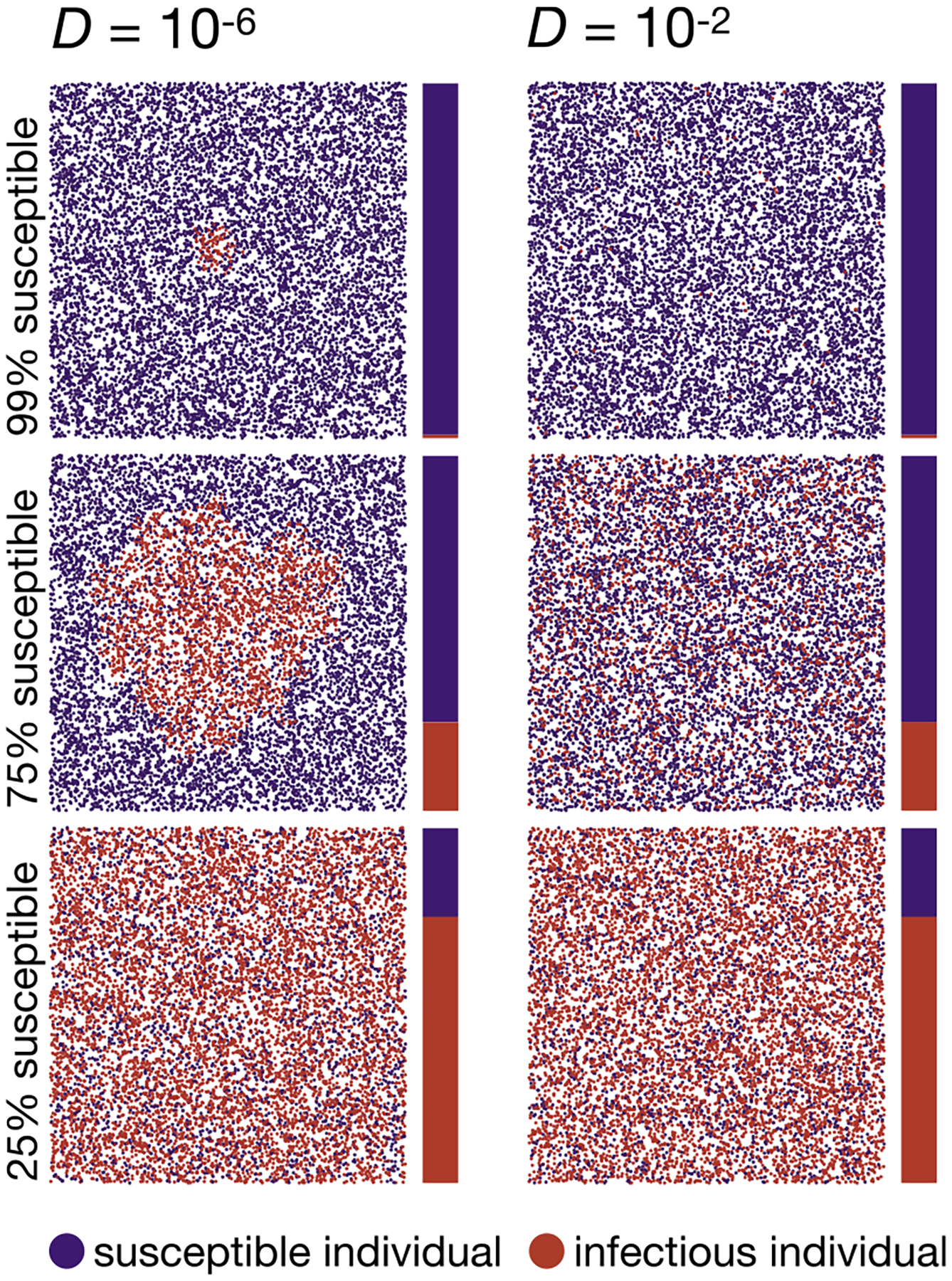
Two example runs of the SIS simulation model. The left column shows a run in the low-dispersal regime ($D=10^{-6}<D_{c}$), while the right column shows a run in the high-dispersal regime $\left(D=10^{-2}\right)$. The top, middle, and bottom plots show population snapshots taken when 99%, 75%, and 25% of the population were still susceptible. As predicted, in the low-dispersal regime, the disease initially progresses in a growing circle. In contrast, in the high-dispersal regime, infectious individuals are homogeneously distributed in space (right column). In contrast to the SI model, in the SIS model, even with low dispersal, infectious individuals hardly cluster at later stages of the simulations due to the continuous recovery of infectious individuals with probability γ per tick. Data were simulated with $D=\left[10^{-6};10^{-2}\right],N=10,000,\alpha =0.01,r=15,L=1,\gamma =1/30$. Videos of simulation runs in the SIS model can be found under https://tinyurl.com/bdddam58.

To investigate the effects of spatial structure and stochasticity on an SIS model, we modified our individual-based simulations so that infected individuals reenter the susceptible class with a probability γ per tick, yielding $R_{0}=r\alpha /\gamma$. Qualitatively, we find that the disease initially still spreads in a circular fashion in the low-dispersal regime and more homogeneously in the high-dispersal regime, as in the SI model (Fig. 4). However, the continuous re-entry of infectious individuals into the susceptible class breaks up this strict circular clustering more quickly in the SIS model than in the SI model. Over the course of the simulated time period, the frequency of infectious individuals converges to the expected constant endemic frequency, $1-1/R_{0}$, in both the high-dispersal and low-dispersal regimes. We note that in our stochastic simulation model with finite population size, this constant endemic frequency is only quasi-stationary: On much longer time scales than considered here, the stochastic model will eventually reach its absorbing, disease-free state (all infectious individuals will eventually disappear from the population).

**Fig. 5.**
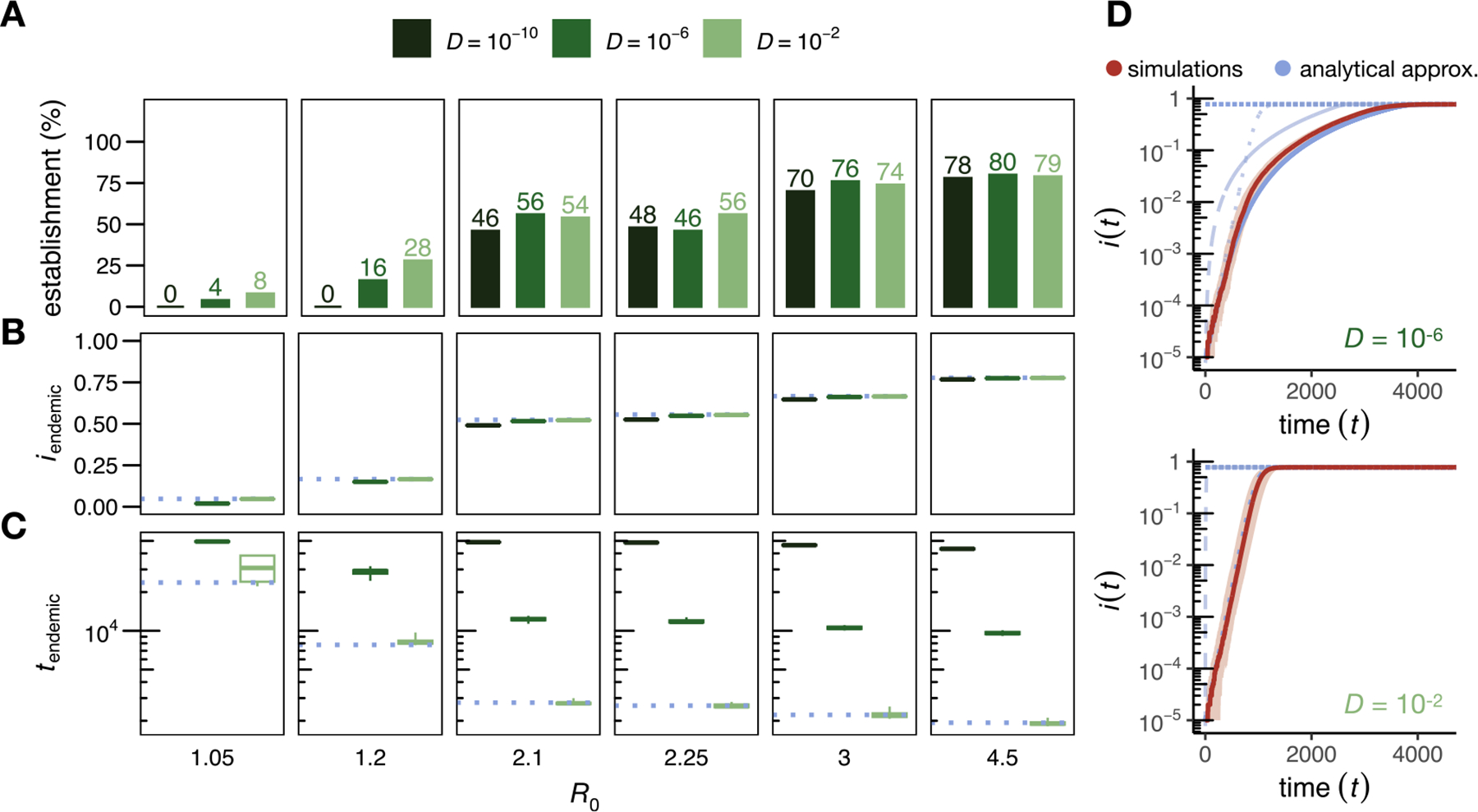
Disease dynamics in the SIS model. (A) Percentage of 50 simulation runs that reached a frequency of at least 1% infectious individuals for different $R_{0}$ values (panels) and three exemplary diffusion coefficients. Only simulation runs with an i(t) of at least 1% are included in (B) to (D). (B) Observed constant endemic frequencies at the end of the simulations. Box plots are shown with whiskers indicating the most extreme values within 5×IQR of the boxes, but the variance between runs was so small that they are not visible. The expected constant endemic frequency for each $R_{0}$ value under an ODE model (Hethcote, 1989) is shown by a dotted blue line. Observed constant endemic frequencies were estimated by fitting simulated data to a three-parameter logistic model using the drc R package (Ritz et al., 2016). (C) Time to reach the constant endemic frequency. We defined the onset of constant i(t) as the first time the frequency of infectious individuals reaches 99.95% of the observed constant endemic frequency. The expected time of constant i(t) onset under an ODE model is depicted as a dotted blue line. (D) Time-resolved disease dynamics for $R_{0}=4.5$ and two exemplary diffusion coefficients (upper panel: low dispersal with $D=10^{-6}<D_{c}=2.87\times 10^{-6}$, lower panel: high dispersal with $D=10^{-2}$). In the low-dispersal regime, the proportion of infectious individuals, i(t), increases after an initial lag phase approximately as the area of a circle whose radius expands at the predicted minimum wave speed of the diffusion model (blue dashed line). The initial lag between the expected and the observed proportion of infectious individuals is likely caused by the local establishment of the disease and an initial reduction in wave speed due to the curvature of the wavefront (Lewis and Kareiva, 1993; Shigesada et al., 1995). The i(t) of the simulations is well approximated by a heuristic approach $t_{\text{fix}}$ (solid blue line) that accounts for both disease propagation via a constant traveling wave, and the local increase in i(t) described by the ODE model (dotted line). In the high-dispersal regime, the proportion of infectious individuals is well approximated by the expected logistic growth function of the ODE model with a carrying capacity of $1-1/R_{0}$ (Hethcote, 1989) (dotted blue line). Solid red lines represent the median proportion of infectious individuals across all simulations, and the shaded area represents the range between the 2.5 and 97.5 percentiles. All data were simulated with N=100,000,α=0.001,r=15,γ=[1/70,1/80,1/140,1/150,1/200,1/300]. More than 99% of our simulation runs categorized as established spread through the entire habitat.

While a deterministic ODE model predicts that any disease with $R_{0}>1$ will establish after introduction, the stochastic fluctuations in our individual-based simulation model can lead to the loss of the disease in certain simulation runs, even for relatively high $R_{0}$ values (e.g., about 25% of simulation runs with $R_{0}=3$, as shown in Fig. 5A). We did not find a strong influence of the dispersal rate on the probability of disease establishment for $R_{0}>2$. However, in the high-dispersal regime, the frequency of infected individuals approaches the expected constant endemic frequency more quickly than in the low-dispersal regime (Fig. 5B–D). Overall, we observed that the rate of disease spread in our simulation model can again differ by more than an order of magnitude, depending on the dispersal rate (Fig. 5C).

### 2.7. SIR model

In the SIS model, individuals transition between the susceptible and infectious states but do not become resistant to the disease. In contrast, in the SIR model, infected individuals acquire permanent resistance and transition to a “recovered state” that cannot be left under the dynamic. In the unstructured SIR model, a disease can invade a fully susceptible population whenever its basic reproduction number $R_{0}=(r\alpha )/\gamma$ is greater than 1 (Hethcote, 1989). In such cases, i(t) first increases to a maximum value $i_{\text{max}}$, then decreases over time, approaching zero as t→∞. In contrast to the SI and SIS models, there is no exact analytical solution for i(t) and approximations are often complex (Bougoffa et al., 2024; Barlow and Weinstein, 2020). However, the maximum incidence $i_{\text{max}}$ can be derived. With I(0)=1 and S(0)=N-1, Hethcote (2000):

(16) $$i_{\text{max}}=1-\frac{\gamma }{r\alpha }\left(1+\text{ln}\;\left[\frac{r\alpha }{\gamma }\frac{N-1}{N}\right]\right)$$

For the time $t_{\text{ODE}}$ to reach this maximum, $i\left(t_{\text{ODE}}\right)=i_{\text{max}}$, a useful approximation has been derived (Eq. 7a Turkyilmazoglu, 2021):

(17) $$t_{\text{ODE}}\approx \frac{1}{r\alpha -\gamma }\;\text{ln}\;\left[\frac{\frac{N-1}{N}+N{\left(\frac{\gamma }{r\alpha }-\frac{N-1}{N}\right)}^{2}}{\gamma /(r\alpha )}\right]$$

Finally, the proportion of all individuals infected at any time during the epidemic, the so-called “final epidemic size”, $e_{\text{final}}$, can be obtained by solving the transcendental equation (Murray, 2002),

(18) $$\text{exp}\;\left[-\frac{r\alpha }{\gamma }e_{\text{final}}\right]=1-e_{\text{final}}.$$

As in the SI and SIS models, we can derive a critical threshold $D_{c}$ for the SIR model, below which individual dispersal affects disease dynamics. We use that the speed of the infection wave in the spatial SIR model is the same as in the spatial SIS model, $c_{0}=2\sqrt{D(r\alpha -\gamma )}$ (Fisher, 1937; Källén, 1984; Wang and Wang, 2016). Thus, the expected time $t_{\text{DIFF}}$ for wave propagation in the spatial SIR model is also equal to the time in the spatial SIS model Eq. (14). Since the infection wave is closely followed by a wave of recovered individuals who cannot be reinfected, the proportion of infectious individuals, i(t), expands approximately as the area of a circular ring of increasing radius and reaches its maximum when this ring reaches the corners of the square habitat patch. As for the SI and SIS models, we obtain a critical value $D_{c}$ of the diffusion coefficient by comparing the expected times required for local growth, $t_{\text{ODE}}$ and for spatial spread, $t_{\text{DIFF}}$, respectively, to reach the maximum $i_{\text{max}}$ of i(t). Specifically, we expect individual dispersal to influence disease dynamics in the spatial SIR model for $D<D_{c}$ with

(19) $$t_{\text{ODE}}=t_{\text{DIFF}}⟹D_{c}=\frac{(r\alpha -\gamma )L^{2}}{8{\left[\text{ln}\;\left(\frac{\frac{N-1}{N}+N{\left(\frac{\gamma }{r\alpha }-\frac{N-1}{N}\right)}^{2}}{\gamma /(r\alpha )}\right)\right]}^{2}}.$$

**Fig. 6.**
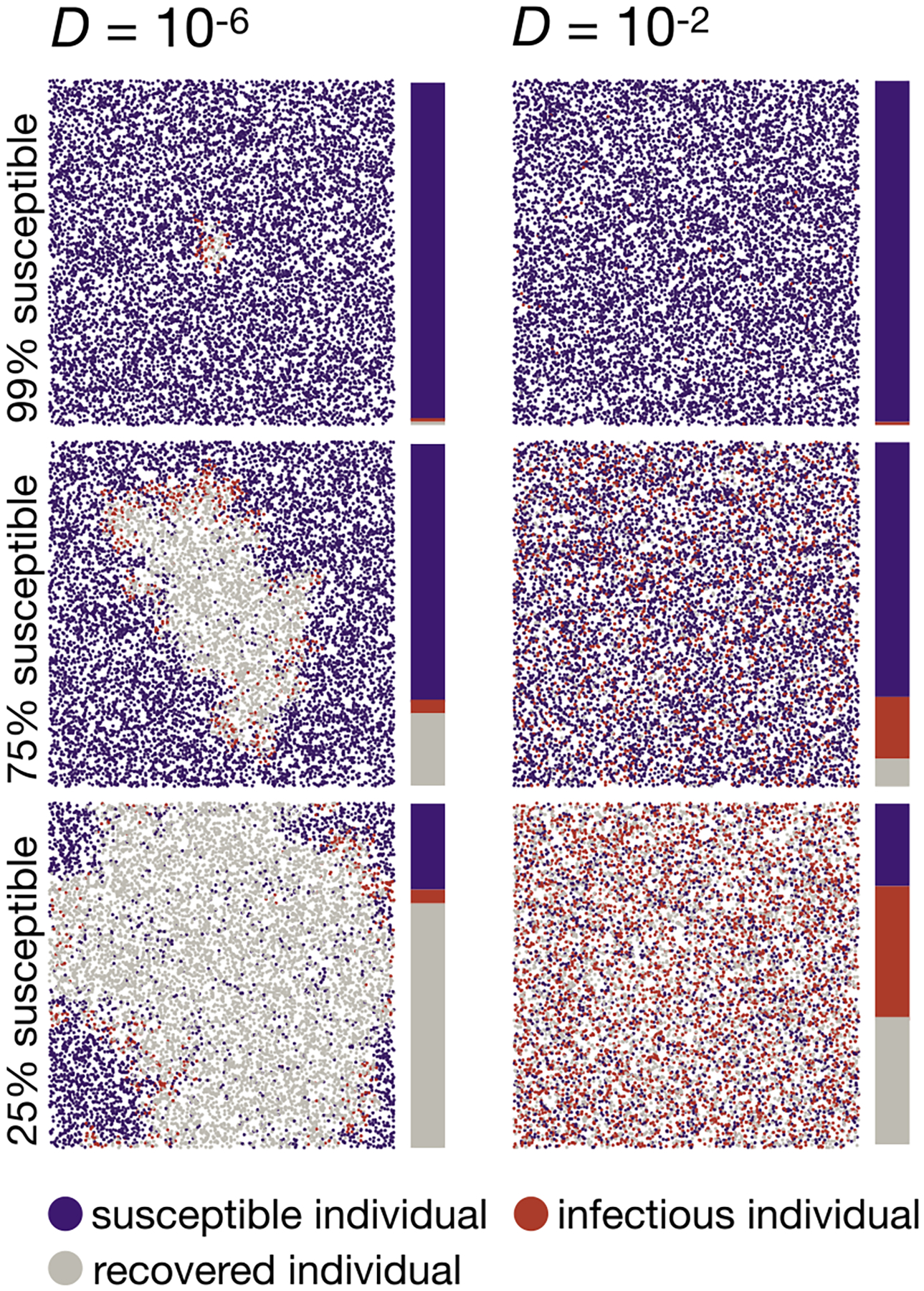
Two example runs of the SIR simulation model. The left and right columns show runs in the low-dispersal ($D=10^{-6}<D_{c}$) and high-dispersal ($D=10^{-2}$) regimes, respectively. The top, middle, and bottom plots show population snapshots taken when 99%, 75%, and 25% of the population were still susceptible. As predicted, in the low-dispersal regime, the disease progresses approximately in a growing circle, with infectious individuals clustered at the wave front, and recovered individuals clustered around the disease origin in the center of the area. In contrast, in the high-dispersal regime susceptible, infectious, and recovered individuals are homogeneously distributed in space (right column). Data were simulated with $D=\left[10^{-6};10^{-2}\right],N=10,000,\alpha =0.01,r=15,L=1,\gamma =1/25$. Videos of simulation runs in the SIR model can be found under https://tinyurl.com/bdddam58.

To study the SIR model, we modified our simulations of the SIS model so that infected individuals transition to the recovered class with a constant probability γ per tick, rather than returning to the susceptible class. This modification does not change the basic reproduction number of the disease, which remains at $R_{0}=r\alpha /\gamma$. Qualitatively, we observed that, similar to the SI and SIS models, the high-dispersal regime closely approximates a homogeneously mixed population (Fig. 6), whereas in the low-dispersal regime, the disease spreads in a circular pattern across space, as theoretically predicted (Cox and Durrett, 1988). However, the traveling wave by which infections spread is now closely followed by another wave describing the emergence of recovered individuals.

**Fig. 7.**
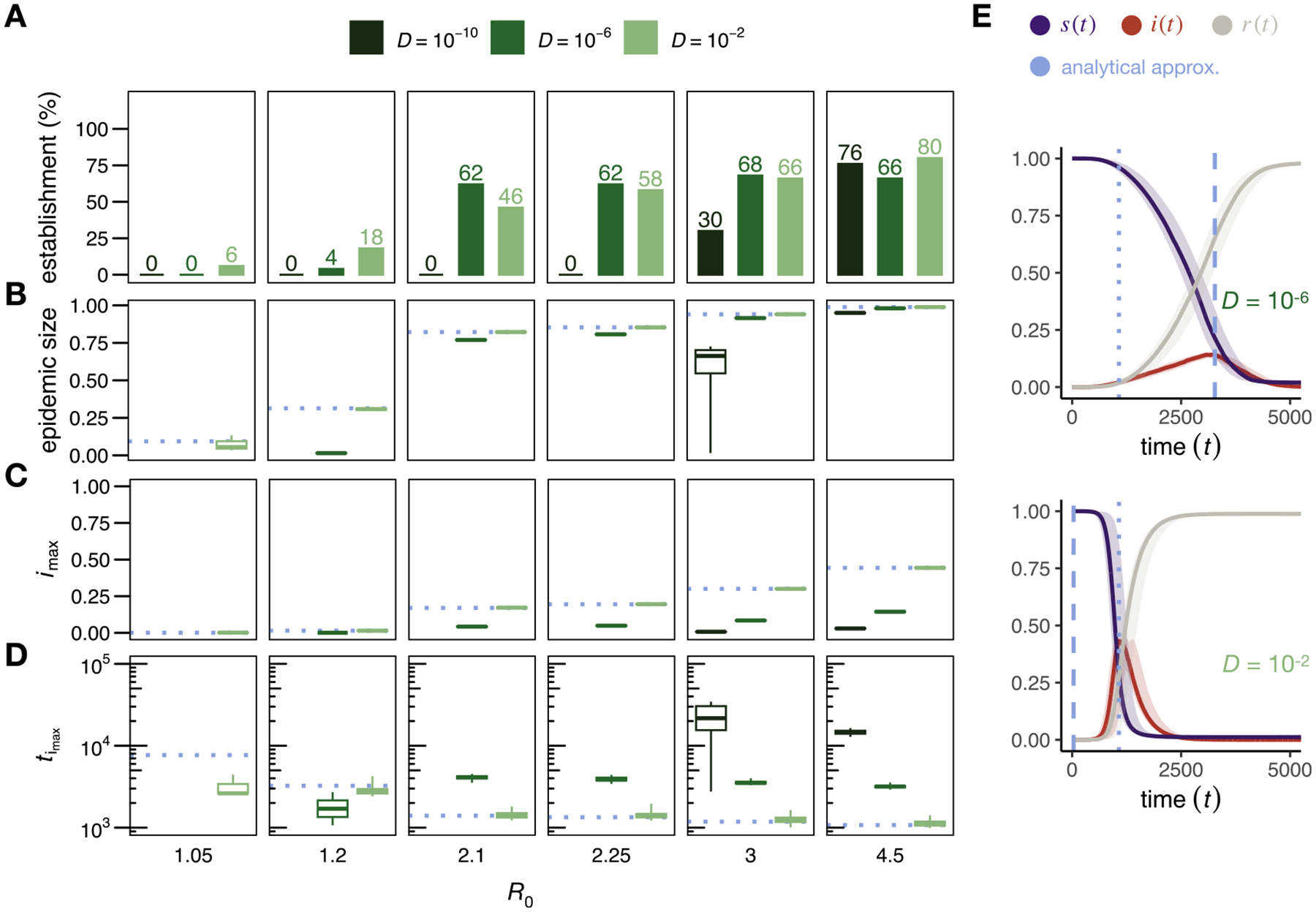
Disease dynamics in the SIR model. (A) Percentage of 50 simulation runs in which the disease reached an epidemic size of at least 1% for different values of $R_{0}$ (panels) and three exemplary diffusion coefficients (for $D=10^{-10}$ and $R_{0}=3$, the spread stagnated in 3 of the 50 runs, which were excluded from the analysis). Disease establishment is less likely in the low-dispersal regime for $R_{0}<2$. Only simulation runs with an epidemic size of at least 1% are included in (B) to (E). (B) Epidemic size (box plots are shown with whiskers indicating the most extreme values within 5×IQR of the boxes). Simulations in the low-dispersal regime require higher $R_{0}$ values to approach the theoretically predicted epidemic size under the ODE model. (C) Maximum incidence $i_{\text{max}}$ (the variance between runs was so small that the whiskers are not visible). The expected $i_{\text{max}}$ (Hethcote, 2000) for each $R_{0}$ value under an ODE model is shown by the dotted blue line. Lower dispersal results in smaller $i_{\text{max}}$ values. (D) Peak Times. Simulations in the low-dispersal regime can take an order of magnitude longer to reach $i_{\text{max}}$ than in the high-dispersal regime. The peak time approximation under an ODE model (Turkyilmazoglu, 2021) is shown as dotted blue line. (E) Time-resolved disease dynamics for $R_{0}=4.5$ and two exemplary diffusion coefficients (upper panel: low-dispersal with $D=10^{-6}<D_{c}=9.31\times 10^{-6}$, lower panel: high-dispersal with $D=10^{-2}$). In the low-dispersal regime, the spatial progression of i(t) can be described as a circular ring whose radius expands with the predicted minimum wave speed and reaches its maximum around the expected peak time in a spatial SIR model (dashed blue line). In the high-dispersal regime, on the other hand, i(t) increases rapidly and reaches its maximum around the expected peak time in an ODE model (Turkyilmazoglu, 2021) (dotted blue line). The solid lines represent the median proportions of susceptible (blue), infectious (red), and recovered (gray) individuals across all 50 simulations, and the shaded areas represent the ranges between the 2.5 and 97.5 percentiles. All data were simulated with N=100,000,α=0.001,r=15,γ=[1/70,1/80,1/140,1/150,1/200,1/300]. More than 99% of our simulation runs categorized as established spread through the entire habitat.

The clustering of recovered individuals close to the wavefront of infectious individuals has several implications for disease dynamics: First, similar to the SIS model, for $R_{0}<2$ there is a higher probability that the disease will be eradicated before it can establish in the low-dispersal regime. This results in a lower probability of establishment for smaller diffusion coefficients. For very weak diffusion ($D=10^{-10}$), the establishment probability remains low even until $R_{0}$ exceeds 3 (Fig. 7A). Second, as previously demonstrated (Grenfell and Dobson, 1995; Mollison, 1991), higher values of $R_{0}$ are required in the low-dispersal regime to approach the theoretically expected final epidemic size (Fig. 7B). If dispersal is limited, the clustering of recovered individuals near the wavefront can lead to isolated ‘pockets’ of susceptible individuals without contact with still infectious individuals. This phenomenon can contribute significantly to reducing the final epidemic size, as illustrated in Fig. 6 for $D=10^{-6}$, where distinct clusters of susceptible individuals surrounded by recovered individuals are observed. Third, because new infections occur only at the wavefront in the low-dispersal regime, the maximum incidence ($i_{\text{max}}$) is smaller than $i_{\text{max}}$ in the high-dispersal regime, which agrees well with the analytical expectations from the ODE model (Fig. 7C). Analogous to the SI and SIS models, reduced dispersal slows down disease spread. Simulations with a diffusion coefficient of $D=10^{-6}$ take almost an order of magnitude longer to reach their respective $i_{\text{max}}$ compared to simulations with $D=10^{-2}$ (Fig. 7D). Finally, the time-resolved frequency of infectious individuals, i(t), resembles a bell-shaped curve in the high-dispersal regime, as predicted by the ODE model (Hethcote, 1989) and the observed peak times agree well with the peak time approximations for the ODE model (Turkyilmazoglu, 2021). In the low-dispersal regime, on the other hand, i(t) shows nearly linear growth until late in the simulation, as predicted by a circular wave progressing at constant velocity (Fisher, 1937; Shigesada et al., 1995; Källén, 1984; Wang and Wang, 2016) (Fig. 7D–E).

## 3. Discussion

In this study, we investigated the conditions under which it is essential to incorporate spatial structure into epidemiological models in order to obtain accurate predictions of disease spread in time and space. We established a critical threshold $D_{c}$ for the diffusion coefficient, below which disease transmission dynamics are expected to exhibit substantial spatial heterogeneity, while for $D\gg D_{c}$ the assumption of homogeneous mixing remains adequate. To validate our analytical results, we performed individual-based simulations on a continuous two-dimensional landscape. Furthermore, we investigated the impact of continuous spatial structure on key epidemiological parameters, including disease establishment probability, maximum incidence, peak time, and final epidemic size.

The use of reaction–diffusion models in epidemiology is attractive because of their ability to approximate epidemic states as individuals move and interact in a spatial domain. They often provide useful estimates for the expected speed of disease propagation based on measurable life-history traits, such as individual dispersal, average contact rate, and infection risk (Hastings et al., 2005). In this study, we focus on the classical Fisher–KPP equation to model diffusive disease transmission because of its simplicity and accessibility to applied modelers. Reaction–diffusion systems with alternative reaction terms and different propagation dynamics have been studied (Lewis et al., 2016) and exact closed-form solutions for traveling wave speeds are available in the literature (e.g., Chen et al., 2012). Exploring alternative reaction terms could lead to broader insights into epidemic spread, but such directions are beyond the scope of this paper.

The Fisher–KPP model assumes that disease transmission is primarily local. Long-range dispersal is rare or absent (Keeling and Eames, 2005; Medlock and Kot, 2003). In addition, distributed-infectives models, as studied here, assume that dispersal is uniform throughout the population, ignoring variations in movement patterns among individuals (Noble, 1974). In reality, individual movement patterns can vary considerably, both in frequency and in direction or distance (Busenberg and Travis, 1983; Reluga et al., 2006). All of these processes can significantly affect the disease dynamics and limit the accuracy of a traveling wave model. For example, an asymmetric dispersal kernel, unlike the radially symmetric one used here, produces an irregular wavefront (Lewis and Kareiva, 1993). Similarly, frequent long-range dispersal, as observed in cases such as wind-dispersed plant pathogens or avian influenza (Shigesada et al., 1995; Shigesada and Kawasaki, 1997; Mundt et al., 2009; Kot et al., 1996; Keeling and Eames, 2005), can cause the disease to spread from multiple locations. In such cases, reaction–diffusion models that assume an exponentially bounded dispersal kernel (Skellam, 1951; Kot et al., 1996; Steiner and Novembre, 2022) underestimate the rate of spread (Shigesada et al., 1995; Kot et al., 1996; Turelli and Hoffmann, 1991; Mundt et al., 2009), complicating efforts to predict and manage outbreaks (Tildesley et al., 2009).

Furthermore, our model assumes constant rates of disease transmission and recovery. In real populations, however, these parameters are often variable. For example, the contact rate r may differ for individuals of different ages (Hastings et al., 2005; Rohani et al., 2010; Mossong et al., 2008) and is typically influenced by landscape features such as major roads, mountains, or rivers (Hastings et al., 2005; Smith et al., 2002). It also varies with population density or changes in behavior (Volz and Meyers, 2007). The probability of disease establishment α is often strongly influenced by environmental factors such as temperature, relative humidity, and the level of urbanization (Eisenberg et al., 2007). All of these factors can play a critical role in shaping the disease dynamics (Lloyd-Smith et al., 2005), yet they are not explicitly included in our current modeling framework.

Finally, in the Fisher–KPP framework, space is a continuous, unbounded domain. This ignores edge effects that exist in real habitats with boundaries (Hotelling, 1921; Fisher, 1937; Hethcote, 2000; Mazzucco et al., 2018). To focus on the effect of individual dispersal on disease spread, without confounding by edge effects, we implemented periodic boundary conditions in our individual-based simulation framework. Reflecting boundaries may better simulate real-world habitats where physical barriers constrain disease spread. However, any resulting boundary effects are highly situational (e.g., depending on the shape of the habitat and the location of the disease outbreak), but generally small in a sufficiently large habitat.

Despite these limitations, there are biological systems that satisfy the assumptions of a reaction–diffusion model with a constant traveling wave. For example, Noble’s analysis of the spread of the Black Death across 14th-century Europe used a reaction–diffusion model, with the predicted spread patterns aligning well with the historical records (Noble, 1974). With parameters rα≈1-4 per year and $D\approx 25,900\;{\text{km}}^{2}$ per year, the distributed-infective process produced a wave “width” of $2\sqrt{D/(r\alpha )}\approx 161-322\;\text{km}$: small relative to the studied region (Messina to Oslo is approximately 3,200 km). Furthermore, long-range dispersal was likely very rare in medieval Europe, supporting the suitability of a constant traveling wave model for studying this particular epidemic. Murray & Brown applied a reaction–diffusion model to potential rabies transmission among foxes in the UK (Murray et al., 1986). Rabies is transmitted by close contact, and foxes are highly territorial, which limits individual dispersal. The model’s rα and D values indicated a small wave width relative to the habitat studied (southern England), with predictions matching rabies spread patterns observed in mainland Europe (Murray et al., 1986).

While it was reasonable to ignore long-range dispersal in the aforementioned studies, the increasingly interconnected nature of modern human populations requires a model that captures both short- and long-range dispersal (Paulose et al., 2019; Kot et al., 1996; Steiner and Novembre, 2022). Integrating these dispersal scales is essential for accurate modeling of human disease spread (Brockmann and Helbing, 2013). For recent human epidemics, Brockmann et al. introduced an “effective distance” measure that accounts for varying levels of connectivity between communities, ultimately allowing the application of relatively simple diffusion models to study complex disease dynamics (Brockmann and Helbing, 2013). However, such connectivity data are rarely available for non-human species, and factors such as behavior, social structures, and environmental conditions can further complicate movement patterns (Steiner and Novembre, 2022; Reiner et al., 2014; Hastings et al., 2005; Brockmann and Helbing, 2013; Lee et al., 2021). For complex movement patterns, network models or individual-based simulations may be more appropriate, although these increasingly complex models also limit the ability to draw general conclusions (Steiner and Novembre, 2022; Hastings et al., 2005).

The critical threshold $D_{c}$ Eq. (9) provides a simple back-of-the-envelope calculation of the amount of individual dispersal required to noticeably slow the disease spread. For example, consider a theoretical measles outbreak in a fully susceptible population. Measles, with up to 90% transmission among close contacts (α=0.9) (Anon, 2022), and assuming four close contacts per day (r=4) (Feehan and Mahmud, 2021), in a population with 979 individuals per km^2^ (i.e., the average population density in urbanized areas of the U.S.) (Anon, 2021), results in $D_{c}=0.002\;{\text{km}}^{2}$ per day. In an area of L=1km, this means that for limited dispersal to significantly slow down the spread of measles, the mean standard deviation of the spatial displacement of individuals in each of the x and y coordinates must be less than 69 meters per day. This toy example shows that spatial dynamics can be neglected for highly contagious diseases over small areas (Kot, 2001), but must be considered for larger communities, such as cities (Grenfell et al., 2001; Chen, 2014).

Our individual-based simulations highlighted important differences and complexities that arise when simulating a continuous reaction–diffusion process. In our discrete framework, we defined contacts between individuals based on an interaction radius parameter, allowing disease to spread by “hopping” between neighboring individuals, even when individuals are stationary. This mechanism imposes an upper limit on the fixation time in our individual-based simulations, which may not be immediately apparent in other disease models. While a k-nearest-neighbor approach could mitigate the influence of interaction radius, caution is warranted when using small k values (e.g., k=1) in combination with low dispersal. Such configurations can substantially slow disease spread and, in some cases, result in coexistence between susceptible and infectious individuals: a phenomenon not predicted by continuous SI or SIR models. Durrett and Levin observed conceptually similar outcomes in their seminal work studying species interactions within spatially distributed populations (Durrett and Levin, 1994). They noted that while continuous models predict the extinction of both species when the two species compete but one cannot survive without the other (analogous to our case where infectious individuals require susceptibles to propagate the infection), discrete models allow for coexistence, particularly in simulation scenarios with low population densities (and thus, reduced inter-species contact rates) (Durrett and Levin, 1994).

In conclusion, our study highlights the need for careful interpretation and understanding of spatial factors in epidemiological dynamics, while also demonstrating the complexities and design choices that arise in modeling such dynamics.

## Data Availability

The individual-based SI, SIS, and SIR disease transmission models are implemented in the open-source software SLiM (version 4.0) Haller and Messer (2023) and are available on GitHub under https://github.com/AnnaMariaL/SpatialDiseaseSim. Videos demonstrating disease spread in structured and unstructured populations are available on YouTube under https://tinyurl.com/bdddam58.
